# Supplementary material for: Genomic and Transcriptomic Survey Provides Insights into Molecular Basis of Pathogenicity of the Sunflower Pathogen Phoma macdonaldii
Source: J Fungi (Basel). 2023 Apr 27;9(5):520. doi: 10.3390/jof9050520 (PMC10218939; doi:10.3390/jof9050520)
Supplement: Supplementary file 1 [file jof-09-00520-s001.zip › Table S1.pdf]

Journal of Fungi

MDPI

Table S1. Primers used in this study

Primer name	Sequence(5′-3′)
Pm_8157-F	TTGTCGCGATTACCCTCTGT
Pm_8157-R	GCGCACAGAAAAGAGTTCCA
Pm_8993-F	CACCAACGCACAACTGATGA
Pm_8993-R	TAGGGCAGAGATCAAGGCAG
Pm_7393-F	GTCATCGTAGGTTGGTTCGC
Pm_7393-R	TGCGTATTGGACCTGGGAAT
Pm_7137-F	ACATGGCCAAGAACGACAAC
Pm_7137-R	GCTCCTCAACTTGTGCTCTG
Pm_7267-F	ATGTGTCTTTTCGTCGGTGC
Pm_7267-R	AGGACAGATTGCCCAACGTA
Pm_9172-F	ATCGCTACCATCTCACTCGG
Pm_9172-R	ACAGATGCAATGCCGATCAC
Pm_5739-F	GCTCGACCGATGGATTATGC
Pm_5739-R	GGTCCTCTTCCAAGTCGTCA
Pm_7268-F	CTGCACACATCCCTGACAAC
Pm_7268-R	TCAGTTGCCGGATACACTGT
Pm_369-F	CCTTTCCATCGTCACCAACC
Pm_369-R	CCCTTCTTGGTGATCTGGGT
Pm_8155-F	AGGATCTGGCAGCATCATCA
Pm_8155-R	TGAACCTGATGACACCAGCT
Pm_10238-F	GCGCTGTTGAACTCGATGAT
Pm_10238-R	CCGTCATAGTCTTTGCTGGC
Pm_8156-F	GATGGAGGCAGCTTGATTGG
Pm_8156-R	CCCAGTCTCCCCATCGATTT
Pm_1474-F	CCCTGGCAACCTAACCTACA
Pm_1474-R	GTGATACTGTGAGCTTCGCG
Pm_1830-F	CCGACTTCCCCTACCTCATC
Pm_1830-R	TCTGTGAGTAGGCCCAGTTG
Pm_8240-F	TTCGTCCCGTCTAGCAACTT
Pm_8240-R	AACAGTCTGGGTCAACGTCT
actin-F	TCACGTCACCACCTTCAACT
actin-R	GTGATCTCCTTCTGCATGCG
