# Supplementary material for: Genomic and Transcriptomic Survey Provides Insights into Molecular Basis of Pathogenicity of the Sunflower Pathogen Phoma macdonaldii
Source: J Fungi (Basel). 2023 Apr 27;9(5):520. doi: 10.3390/jof9050520 (PMC10218939; doi:10.3390/jof9050520)
Supplement: Supplementary file 1 [file jof-09-00520-s001.zip › Table S10.pdf]

Journal of *Fungi*

**Table S10.** Summary statistics for the Illumina sequencing and mapping against *P. macdonaldii* CXJ0811 reference genome.

| **Sample** | **Number of raw reads** | **Number of reads after trimming** | **Number of mapped reads on *P. macdonaldii* CXJ0811 genome** | **% of mapped reads on *P. macdonaldii* CXJ0811 genome** |
|---|---|---|---|---|
| CT | 44,502,032 | 43,419,308 | 41,415,549 | 95.39% |
| LEAF-2d | 37,670,824 | 37,209,286 | 23,236,530 | 62.45% |
| LEAF-6d | 81,178,668 | 78,841,080 | 385,245 | 0.49% |
| STEM | 36,287,822 | 34,809,958 | 31,822,798 | 91.42% |
